# Identification of an atypical replicative genetic element in *Rhodococcus jostii* RHA1

**DOI:** 10.3389/fmicb.2025.1567901

**Published:** 2025-06-02

**Authors:** Miguel G. Acedos, Isabel de la Torre, Jorge Barriuso, José L. García

**Affiliations:** ^1^Department of Biotechnology, Centro de Investigaciones Biológicas Margarita Salas, Consejo Superior de Investigaciones Científicas (CSIC), Madrid, Spain; ^2^Advanced Biofuels and Bioproducts Unit, Department of Energy, Centro de Investigaciones Energéticas, Medioambientales y Tecnológicas (CIEMAT), Madrid, Spain

**Keywords:** *Rhodococcus*, kanamycin, illegitimate recombination, replicative genetic element, antibiotic resistance

## Abstract

By exploring the use of plasmids to confer *Rhodococcus jostii* RHA1 the possibility of utilizing xylose to produce lipids we have observed that the plasmid used was not always maintained in the transformants as expected. Instead, we observed an illegitimate integration of the antibiotic resistance gene from the plasmid into the recombinant cells. Genome sequencing of the transformants has provided evidence that this illegitimate integration is not size-, site-or sequence-specific. But even more surprising, genome sequencing revealed the presence of an unexpected circular multicopy replicative element (75–80 kb) that appears to be excised from the chromosome as a consequence of the stress generated by the antibiotic used in the selection process. The excised fragment does not contain any of the typical features of genomic islands. These results provide evidence that the genome of this oleaginous strain is more plastic than initially anticipated and our findings open the option of developing new ways to genetically modify this strain by using illegitimate recombinant approaches. But even more remarkably, the discovery of this atypical replicative element raises new questions about the existence of novel mechanisms of evolution in bacteria.

## 1 Introduction

*Rhodococcus* is a bacterial genus belonging to the phylum Actinobacteria that comprises a large number of genetically and physiologically diverse species that have been studied for their application in biotechnological processes (Hernández et al., 2008; Donini et al., 2021; Li et al., 2021). In this sense, some *Rhodococcus* species are able to accumulate significant amounts of lipids, above 20% of the cell dry weight, and are referred to as “oleaginous bacteria” and used as source of biofuels (Álvarez et al., 2021). In particular, *Rhodococcus opacus* PD630 and *Rhodococcus jostii* RHA1 strains are considered oleaginous models since they produce large intracellular amounts of triacylglycerols (TAGs) reaching up to 80% of the cell dry weight (Voss and Steinbüchel, 2001; Fei et al., 2015; Herrero et al., 2018). To improve the lipid production capacities of these two microorganisms, different genetic modifications have been done (Ding et al., 2012; Villalba and Álvarez, 2014; Huang et al., 2016; Hernández and Álvarez, 2019; Kim et al., 2019). In particular, much attention has been paid to the use of lignocellulosic biomass as feedstock for the production of TAGs. Some recombinant strains have been engineered to assimilate pentoses, such as xylose, and produce lipids (Hetzler et al., 2013; Xiong et al., 2012, 2016a, 2016b; Hernández et al., 2015; Kurosawa et al., 2013).

Interestingly, both strains have one of the largest bacterial genomes sequenced to date, arranged in a linear chromosome and several linear plasmids (McLeod et al., 2006; Firrincieli et al., 2022). The engineering of *Rhodococcus* is still challenging because of the high genome GC content (61% ~ 71%), a low transformation and a limited number of available genetic engineering tools (Liang and Yu, 2021). Moreover, when transforming *Rhodococcus* we have to consider the advantages or disadvantages that homologous or illegitimate recombination entail (DeLorenzo et al., 2018; Desomer et al., 1991; Kim et al., 2019; Kitagawa et al., 2001; Liang and Yu, 2021; Ma et al., 2010; Round et al., 2019; van der Geize et al., 2000, 2001, 2008; Yano et al., 2015).

In this work, we have generated a genetic construct to confer *R. jostii* RHA1 the ability to metabolize xylose, as well as to increase the lipid production yield by overexpressing a gene to facilitate the fatty acid incorporation into TAGs. The aim was to create a stable plasmid, however, the RHA1 strain showed a particular behavior since the plasmid was not always maintained in the host as expected. Instead, we observed an illegitimate integration of the plasmid region containing the kanamycin resistant gene in the chromosome of the transformed cells and even more surprising, the presence of a circular multicopy replicative element excised from the chromosome that behaves as an atypical genomic island. We hypothesize that the creation of this circular element is most probably done to reduce the stress generated by the high concentration of kanamycin used in the selection process that cannot be fully alleviated by the expression of the single copy of the integrated kanamycin resistant gene. This result provides evidence that this oleaginous bacterial strain presents higher genetic plasticity than initially anticipated, opening not the option to develop new ways to genetically modify this strain through the use of illegitimate recombinant protocols, but also showing that this strain possesses peculiar adaptative mechanisms to evolve responding to stress conditions.

## 2 Materials and methods

### 2.1 Strains, plasmids and culture conditions

The pNVs *Escherichia coli-Rhodococcus* shuttle vector (Guevara et al., 2017) (Supplementary Figure S1) and *R. jostii* RHA1 strain were kindly provided by Dr. J. M. Navarro-Llorens from the University Complutense of Madrid (Spain). *Escherichia coli* DH5α from Thermo Fisher Scientific was employed for cloning experiments.

*E. coli* DH5α was cultured at 37°C in LB medium (Felpeto-Santero et al., 2015). *Rhodococcus* strains were cultured at 30°C on minimal medium W or LB medium. Minimal medium W per liter consisted of 0.85 g KH_2_PO_4_, 4.90 g Na_2_HPO_4_ 12H_2_O, 0.50 g (NH_4_)_2_SO_4_, 0.10 g MgSO_4_ 7H_2_O, 9.50 mg FeSO_4_ 7H_2_O, 10.75 mg MgO, 2.00 mg CaCO_3_, 1.44 mg ZnSO_4_ 7H_2_O, 1.12 mg MnSO_4_ 4H_2_O, 0.25 mg CuSO_4_ 5 H_2_O, 0.28 mg CoSO_4_ 7 H_2_O, 0.06 mg H_3_BO_4_, and 5.13 × 10^−2^ mL concentrated HCl. Sterilized stock solutions of xylose (30 g/L), were filtered through a 0.22-μm pore size filter and then added to the autoclaved medium. When required kanamycin was added to the culture medium at 150 μg/mL. Agar plates were prepared adding 13% agar to the media.

### 2.2 Construction of recombinant strains

A *xylABatf1* synthetic operon was designed to facilitate the expression of the following genes: *xylA* (xylose isomerase) and *xylB* (xylulokinase) from *Streptomyces lividans* TK23, and *atf1* (acyltransferase) from *R. opacus* PD630 (Xiong et al., 2012, 2016a, 2016b). These genes were cloned into the pNVs shuttle vector, under the control of the *Ptac* promoter. To enhance mRNA translation efficiency, a consensus Shine-Dalgarno sequence (AGGAGG) was incorporated upstream of each gene, positioned 6 bp from their respective start codons. Codon usage was tailored to *Rhodococcus* using the Optimizer software by Genescript. The *xylABatf1* operon (Supplementary Figure S2) synthesized by Genescript was cloned into the pNVs vector through the *Spe*I and *Pac*I restriction sites rendering plasmid pNVxylABatf1 (Supplementary Figure S3).

Competent *E. coli* DH5α cells were prepared using CaCl_2_ and subjected to transformation through the heat shock procedure (Tang et al., 1994). Competent *Rhodococcus* cells were prepared as follows: cells were cultured in LB medium until reaching an optical density at 600 nm (OD_600_) of 0.5–0.6, were then harvested, washed with water, resuspended in 10% glycerol, and subsequently electroporated with plasmids-Electroporation was performed using a Gene Pulser (Bio-Rad) eletroporator equipment at 400 Ω, 25 mA, and 2.5 μF. Immediately following electroporation, cells were transferred to 1 mL of LB and the expression process was carried out in 10 mL plastic tubes for 6 h at 30°C, without agitation. Thereafter, cells were incubated on LB agar plates supplemented with kanamycin (150 μg/mL) at 30°C to select the kanamycin resistant clones.

### 2.3 DNA extraction, genome sequencing and semiquantitative PCRs

Plasmid DNA was extracted using the QIAwave Plasmid Miniprep Kit (Qiagen) according to the manufacturer’s instructions. Briefly, bacterial cultures were grown overnight in LB medium at 37°C with shaking. The cells were harvested by centrifugation, and the plasmid DNA was isolated using the kit’s plasmid isolation protocol, which includes cell lysis, neutralization, and subsequent purification through silica membrane columns. The plasmid DNA was eluted in an appropriate volume of elution buffer and quantified using a spectrophotometer.

Total DNA extraction of *R. jostii* RHA1 wild type and transformed strains was performed as described (Uhía et al., 2011). The whole-genomes were obtained by Illumina sequencing and were assembled *de novo* by Microbes NG^1^ using its standard pipeline. The raw reads were also mapped to the reference genome of *R. jostii* RHA1 (NCBI RefSeq assembly GCF_000014565.1) using Geneious v2020.0 software. Additional data about sequencing and assembling procedures are provided in Supplementary material.

Semi-quantitative PCR was performed to corroborate the high copy number of the chromosomal excised circular element using two primers inside the genomic island named iGIforward (GCCAGTTTCACCATCGACCA) and iGIreverse (GTTGTCGAACACGGCCAC) corresponding to RHA1_RS22285 (4,822,607–4,822,626) and RHA1_RS22290 (4,822,789–4,822,806) respectively; and two primers outside the genomic island named oGIforward (GCTCTTCGACAGGTACCGC) and oGIreverse (GCCCTCGTTGCGCAGTAG) corresponding to RHA1_RS29475 (6,485,280–6,485,298) and RHA1_RS29475 (6,485,452–6,485,569). PCR amplification was carried out with *Taq* DNA Polymerase (New England Biolab) using 18 cycles and a template DNA concentration of 100 ng/μL.

## 3 Results

### 3.1 Non-homologous recombination in *Rhodococcus*

The primary aim of this work was to allow *R. jostii* RHA1 to grow using xylose as sole carbon and energy source. To fulfill this aim *R. jostii* RHA1 cells were electroporated with pNVxylABatf1 plasmid and as expected, many transformant colonies resistant to kanamycin were obtained. However, when these colonies (20 colonies) were isolated and the presence of pNVxylABatf1 plasmid was analyzed by plasmid DNA extraction of a liquid culture, in at least 80% of them we did not detected the plasmid even though the cells were cultured in liquid LB medium containing 150 μg/mL of kanamycin. This result suggested that: (i) the plasmid copy number has decreased drastically and it was not possible to detect it by conventional plasmid extraction procedures; (ii) kanamycin resistance gene has been integrated into the chromosome, or (iii) the cells are able to mutate and become kanamycin resistant by a different mechanism.

To verify the third hypothesis, we screened on kanamycin containing plates the apparition of kanamycin resistant colonies after subjecting cells of *R. jostii* RHA1 to electroporation in the absence of plasmid or just by plating the native strain without having been subjected to electroporation. In all these experiments, we were not able to obtain colonies resistant to kanamycin, discarding the hypothesis that *R. jostii* can acquire spontaneously the resistance to kanamycin with or without electroporation. This result indicates that kanamycin resistance was conferred by the kanamycin resistance gene harbored by pNVxylABatf1 plasmid.

**Figure 1:**
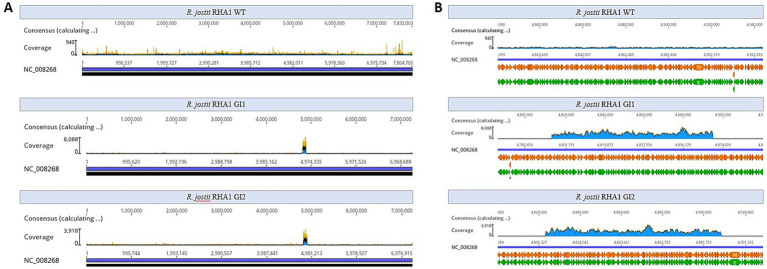
Illegitimate recombination of plasmid pNVSxylABatf1 in the chromosome of *R. jostii* RHA1. **(A)** Schematic description of recombination observed in clone 1. **(B)** Schematic description of recombination observed in clone 2. Green arrows represent the genes located in the *R. jostii* chromosome that are deleted after recombination: (1) LuxR C-terminal-related transcriptional regulator; (2) acyl--CoA ligase family protein; (3) DUF3054 domain-containing protein; (4) TetR/AcrR family transcriptional regulator; (5) flippase-like domain-containing protein; (6) YceI family protein; (7) TetR/AcrR family transcriptional regulator; (8) Alpha/beta hydrolase. The blue arrows represent the genes located in plasmid pNVSxylABatf1 that are inserted after recombination. The orange triangles represent the recombination sites in the chromosome of *R. jostii* marked as I and II for clone 1 and as V and VI for clone 2, and in the plasmid pNVSxylABatf1 marked as III and IV for clone 1 and as VII and VIII for clone 2. Numbers in the sequences indicate the position at the *R. jostii* chromosome. The sequences surrounding the insertion sites both in the chromosome and in the plasmid are indicated.

To determine if the kanamycin resistance gene was integrated in the genome, we sequenced the complete genomes of two electroporated kanamycin-resistant clones (named clone 1 and clone 2) in which we were unable to detect the pNVxylABatf1 plasmid. We also sequenced the genome of the wild type strain as control. These analyses revealed that the kanamycin resistance gene together with a small fragment of the pNVxylABatf1 plasmid was located in clone 1 inserted in the genome between positions 244,546 and 245,979 of the chromosome, generating a deletion of 1,434 bp in the LuxR C-terminal-related transcriptional regulator (locus_tag: RHA1_RS00970) and 27 terminal nucleotides of acyl-CoA ligase family protein (locus_tag: RHA1_RS00975) genes (Figure 1). In the case of clone 2, the kanamycin gene together with a different small region of the pNVxylABatf1 plasmid was inserted in the genome between positions 5,497,080 and 5,500,601 of the chromosome, generating a deletion of 3,522 bp in the DUF3054 domain-containing protein (locus_tag: RHA1_RS25245), TetR/AcrR family transcriptional regulator (locus_tag: RHA1_RS25250), flippase-like domain-containing protein (locus_tag: RHA1_RS25255), YceI family protein (locus_tag: RHA1_RS25260), TetR/AcrR family transcriptional regulator (locus_tag: RHA1_RS25265) and the last 6 nucleotides of alpha/beta hydrolase (locus_tag: RHA1_RS25270) genes (Figure 1). As expected the genome sequence of the wild type strain was identical to the reference genome (NCBI RefSeq assembly GCF_000014565.1) confirming that kanamycin resistance was acquired through specific insertion and deletion processes.

The comparison of the integration sites observed in both clones suggests that the integration is not site specific. This analysis also indicates that it has occurred through a non-homologous recombination mechanism, since we have not found homologous sequences between the plasmid sequences and the *Rhodococcus* genome. Moreover, we have not found palindromic or repetitive sequences at both ends of the integration sites that could suggest a sequence specific recombination mechanism.

### 3.2 Presence of a circular genetic element in *Rhodococcus*

**Figure 2:**
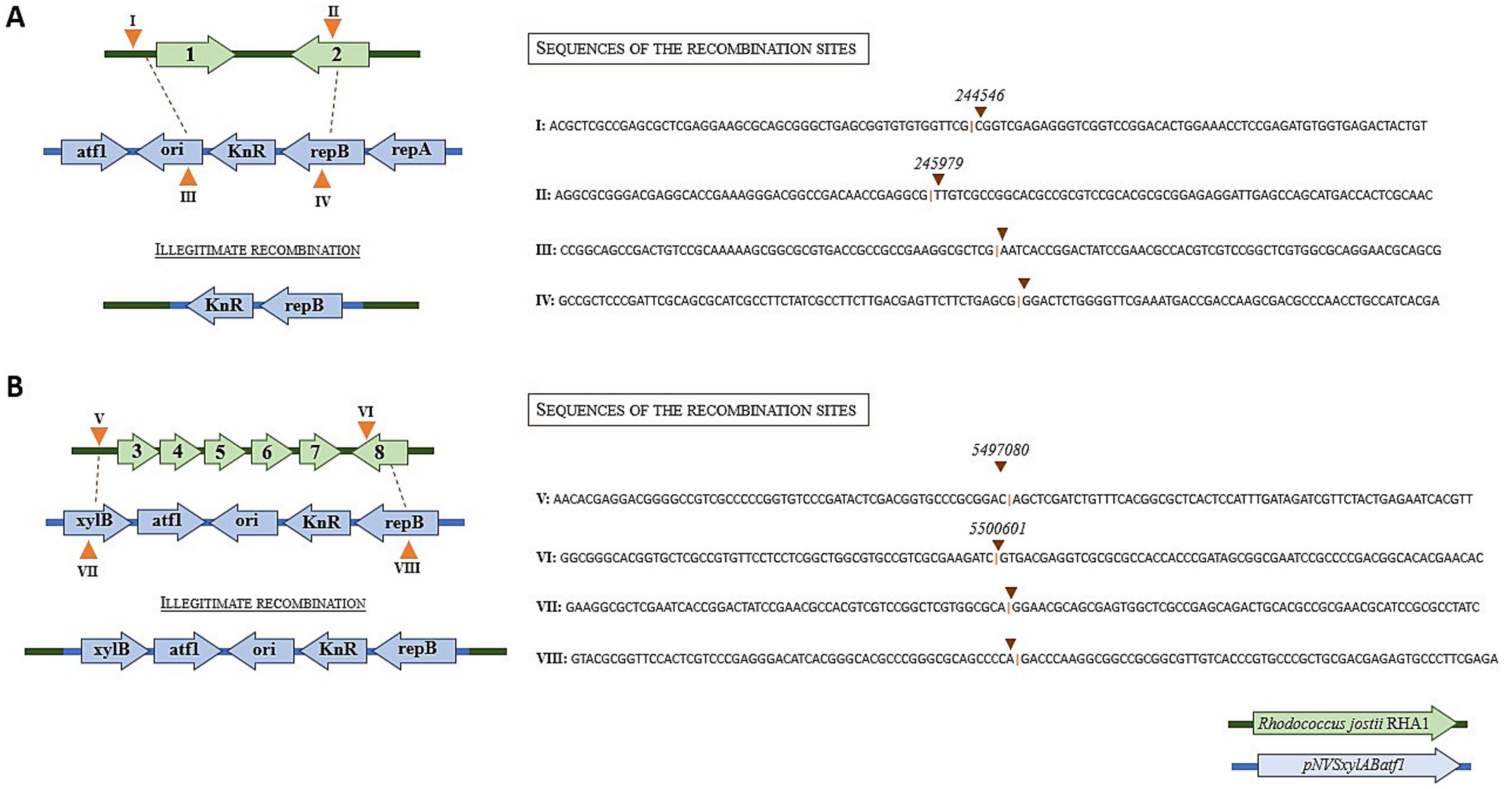
Coverage of the reads sequenced by Illumina. **(A)** Reference genome of *Rhodococcus jostii* RHA1, showing alignment of the genomes of the two transformed microorganisms, with the 80 kb excised fragment clearly visible, indicated by a notable increase in read coverage. **(B)** Zoom of the genomic island region with high coverage in clones 1 and 2. The zoom also highlights the region with high coverage in the WT strain, which does not present the excised element.

A detailed analysis of the assembled contigs of the two kanamycin resistant clones revealed that both genomes contained a large contig of 75 kb in clone 1 and 82 kb in clone 2, covering the same region with an anomalous high coverage of around 1,400 and 800 in clones 1 and 2, respectively, when compared with the coverage of the other chromosomal contigs that were around 40 and 30 in average in clone 1 and 2, respectively. The coverage of this large contig was also larger than that of the plasmids present in this strains pRHL1, pRHL2 and pRHL3 that showed average coverages of 87, 95, and 107, respectively, in both clones. The high coverage of this contig can be easily observed when the reads were aligned to the reference chromosome (Figure 2). The coverage of this region in wild type strain is average (i.e., 30–40 coverage) with the other contigs in the assembled genome. Thus, the anomalous high coverage of the large contig of clone 1 and 2 suggests that this region has been replicated about 38-fold in clone 1 and about 25-fold in clone 2.

A detailed analysis of the assembled genomes of clones 1 and 2 revealed that although both high copy number contigs cover the same genomic region they were not identical. The contig of clone 1 extends from position 4,795,129 to position 4,869,985, and the contig of clone 2 extends from position 4,808,336 to position 4,890,131 (Figure 2). The genes encoded in these contigs are shown in Supplementary Tables S1, S2. The annotation of this region did not identify the presence of recombinases or integrases that are typically found within and at the ends of mobile elements. Moreover, we were unable to annotate Rep proteins inside the region.

The analysis of the whole reads in both clones 1 and 2 identified many reads containing the 5′ and 3′ ends of the element supporting the fusion of 5′ and 3′ ends of the high coverage contig, suggesting a circular structure of the sequenced contig. The possibility that these specific reads can be generated by the precise and consecutive copy of this region many times in the genome cannot be completely discarded, but it is very unlikely. Large genomic regions can be duplicated or triplicated in a genome (Raeside et al., 2014), but they cannot be consecutively multiplied 20 to 30-folds as suggested by the values of the coverage. This is not an artifact of DNA sequencing since the high coverage of this contig was not detected in the genome of wild type strain.

Considering that we have found reads that span both the original linear region of the plasmid and circular reads of the excised element, this result suggests that this genomic region is still integrated in the chromosome. Therefore, the large number of copies of the region released from the chromosome must be due to a replicative event. The putative integration sites are different in both clones and have not repetitive sequences. This observation suggests that the region is excised from the genome and it is further maintained and amplified as a circular DNA structure. It is worth to mention that in clone 1 the circularisation of the region is produced by three identical nucleotides (GCT) located at both ends of the regions named whereas in the case of clone 2 the circularization is produced by four identical nucleotides (GGCC) located at both ends of the region that are part of a non-identical 6-bp palindromic sequences (Supplementary Figure S3). It is also surprising that when aligned the regions A and C, and the region B and D surrounding the excision left and right sites of the element we found a partial reverse complementarity (Supplementary Figure S3). In addition, the GC content of the excised fragment does not show any difference with the GC content of whole genome or even with the GC content of the surrounding sequences that can suggest a recent horizontal transfer.

Whereas the excision of the region from the genome can be explained by the action of recombinases present outside of the element, the high copy number of the resulting circular element should be explained by a replicative mechanism. In this sense, this DNA region does not contain putative proteins involved in DNA replication that might facilitate the identification of a putative *ori*. Several genes encoding DNA replicative functions that could be used to replicate the element have been described in the chromosome and in the three plasmids of RHA1 (McLeod et al., 2006), but it is worth to mention that the chromosome and plasmids of this strain are linear. Therefore, the mechanism used to replicate this excised fragment is unknown.

**Figure 3:**
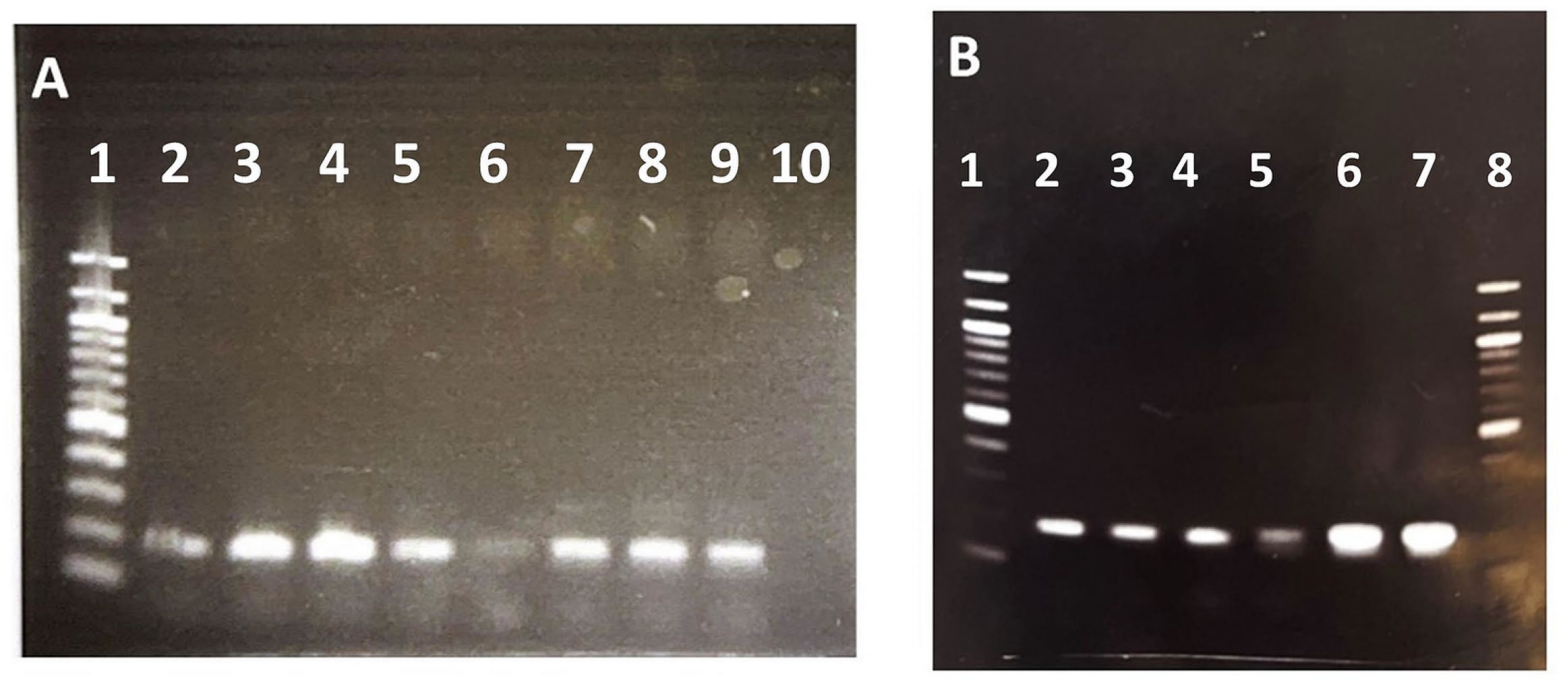
Semiquantitative PCR of different *R. jostii* kanamycin resistant clones electroporated with pNVS plasmid. **(A)** PCR amplification performed with primers iGIforward and iGIreverse inside the mobile region. Lane 1. 1 kb DNA Ladder. Lanes 2, 3, 4, 5, 7, 8, 9. Km resistant clones; Lane 6. Wild type. Lane 10. Negative control. **(B)** PCR amplification performed with primers oGIforward and oGIreverse outside the mobile region. Lane 1. 100 bp DNA Ladder. Lanes 2. Wild type. Lane 3 Clone 1 (sequenced). Lane 4. Clone 2 (sequenced). PCR amplification performed with primers iGIforward and iGIreverse inside the mobile region. Lane 5. Wild type. Lane 6. Clone 1 (sequenced). Lane 7. Clone 2 (sequenced). Lane 8. 100 bp DNA Ladder.

Finally, to determine if the amplification of this specific region was also present in more kanamycin resistant clones we analyzed by semiquantitative PCR the genomes of these colonies. Figure 3A shows that the intensity of the band corresponding to this region is higher in other kanamycin-resistant colonies when compared with the band observed in wild type strain, suggesting that the amplification was associated to the acquisition of kanamycin resistance. In addition, to confirm the amplification of the mobile region the genomic DNA extracted from the sequenced clones 1 and 2 and wild type strain were amplified with two pairs of primers selected inside and outside the mobile region. Figure 3B shows that as expected the intensities of PCR bands outside the region are similar in wild type and clones 1 and 2, whereas the intensity of PCR band inside the mobile region of wild type is very low when compared to the intensities of the PCR bands obtained with clones 1 and 2.

## 4 Discussion

In our attempts to transform *R. jostii* RHA1 with the plasmid pNVxylABatf1 we observed that only few kanamycin resistant colonies were able to maintain a stable kanamycin resistant phenotype. Most of the kanamycin resistant clones obtained after electroporation do not contain the pNV plasmid suggesting that the kanamycin resistance of these clones has been acquired by integration of the kanamycin resistance gene. In this sense, Desomer et al. (1991) have demonstrated that the electrotransformation of *Rhodococcus fascians* with non-replicating plasmids containing an antibiotic resistance marker resulted in stable transformants by illegitimate recombinant integration of these constructs at different sites in the genome, thereby generating different mutations that can be useful as a genetic mutational tool. Genetic toolkits based on the same principle of illegitimate recombination have been described for *Rhodococcus* ssp. (Liang and Yu, 2021). Interestingly, when Kitagawa et al. (2001) used a kanamycin resistance gene derived from Tn903, to select homologous recombination events in *R. jostii* RHA1 all the transformants had insertions at loci other than the original gene locus, demonstrating that illegitimate recombination had occurred, and suggesting that the kanamycin resistance gene derived from Tn903 may contain a sequence that promotes illegitimate recombination. Moreover, van der Geize et al. (2000) also described insertional inactivation of the *kstD* gene from *R. jostii* RHA1 in response to the presence of a kanamycin resistance gene derived from Tn5. Nevertheless, illegitimate recombination was observed using other antibiotic resistance markers in *R. opacus* PD630 a strain closely related to *R. jostii* RHA1 (DeLorenzo et al., 2018) suggesting that non-homologous recombination is not strictly dependent of the resistance marker. Although most of the experiments that have demonstrated illegitimate recombination have been performed with antibiotic resistance markers due to their easy selection, this does not mean that illegitimate recombination is only linked to the selection pressure induced by these markers.

In agreement with these previous demonstrations that illegitimate recombination is a process that can be frequently observed in *Rhodococcus*, our results provide a highly precise demonstration based on genome sequencing that *R. jostii* RHA1 is able to become kanamycin resistant by the integration of the heterologous kanamycin gene by non-homologous recombination. In the two sequenced clones 1 and 2, the integration of the kanamycin marker gene took place in two different sites of the genome and moreover, the integrated regions were also different in both clones dragging different surrounding sequences of the plasmid. We have not detected specific sequences that could explain the integration, suggesting that they occurred by illegitimate recombination events and that they cannot be ascribed to specific sequences present in the kanamycin resistance gene or in the plasmid. In our case, the illegitimate recombination of the kanamycin resistance gene was not forced by the transformation with linear fragments or non-replicative plasmids, since it occurred using a known replicative plasmid, that in fact can be isolated as such only in 20% of our transformants. Why pNVxylABatf1 plasmid can be maintained only in some transformants is not known, but we hypothesized that only transformant that can maintain a large copy number of the plasmid are able to support the high kanamycin concentration that we use in the agar plates. In fact, we have observed that a large amount of plasmid can be extracted from the colonies where plasmid remains stable supporting the hypothesis that only the colonies that have a large copy number of this plasmid can support the kanamycin pressure (data not shown).

It is known that integration by non-homologous recombination can occur mediated by tyrosine recombinases (Grindley et al., 2006; Rajeev et al., 2009) and in this sense, the genome of *R. jostii* RHA1 encodes at least 26 recombinases and 20 integrases (Supplementary Table S3), that could explain the indels observed in the sequenced clones 1 and 2.

All the above results perfectly agree with the existing literature on the illegitimate integration of marker sequences in *Rhodoccocus* ssp., however when we analyzed the genomes of clones 1 and 2 to confirm the integration of the kanamycin resistance gene, unpredictably, we observed that the integration correlates with a great amplification of a specific large genomic region located very far from the marker integration sites. The sequencing data suggest that the amplification of this region generate a circular DNA structure similar to those structures generated by the amplification of Genomic Islands (GI).

GIs are usually large, but discrete and unstable segments of chromosomal DNA that can encode mobility functions enabling their intra-and/or inter-cellular mobility. They can provide adaptive functions enhancing the bacterial fitness and survival. GIs can encode different functions including resistance to toxic compounds, pathogenicity and toxins factors, colonization traits, and alternative metabolic pathways, among others (Juhas et al., 2009; Izumiya et al., 2011; Levy-Booth et al., 2019). Horizontal gene transfer facilitated by GIs has played a crucial role in the evolution of bacterial species. GIs are usually found in specific subsets of closely related strains, which is a key indicator of their acquisition through horizontal gene transfer (Juhas et al., 2009). However, it has been shown that genomic islands are also formed to acquire new metabolic capabilities, resistance or adaptation to new environments (van der Meer and Sentchilo, 2003; Pathak et al., 2016). The term GI involves several types of mobile genetic elements (MGEs) with various structures and gene contents, including prophages, transposons, integrated plasmids, integrative and mobilizable elements (IMEs), and integrative and conjugative elements (ICEs) (Bellanger et al., 2014; Johnson and Grossman, 2015; Audrey et al., 2023). The name ICE was initially proposed by Burrus et al. (2002) for a diverse group of MGEs, which have both plasmid-and bacteriophage like features. ICEs are present in all major divisions of bacteria and include GIs and conjugative transposons. Actinomycetes have specific integrative and conjugative elements named AICEs that are characterized by their prophage-like mode of maintenance, i.e., replication along with the host chromosome, and their ability to excise, conjugate to a new host and integrate in the host chromosome by site-specific recombination, irrespective of the specificity and mechanism of integration and conjugation (te Poele et al., 2008). For AICEs, the DNA is translocated as a double-stranded molecule by a DNA translocase of the FtsK/SpoIIIE family (Choufa et al., 2022). The discovery and earliest studies of ICEs resulted from interest in resistances to antibiotics and heavy metals, and how those resistances were spread between organisms (Johnson and Grossman, 2015). Many of the putative ICEs that have been identified bioinformatically are likely to have cargo genes with functions distinct from those already associated with most well characterized ICEs. Understanding the function of these cargo genes can reveal important information about the specific ICE, its host, and the environment in which the current and/or previous host normally resides. ICEs can replicate autonomously when they are induced and excised from the chromosome, increasing the copy number of all ICE genes, but not of the adjacent chromosomal genes (Johnson and Grossman, 2015). Imprecise excision of an ICE, as observed in our case, is not infrequent (Johnson and Grossman, 2015) and can occurs bringing along flanking genes, analogous to the imprecise excisions that generate transducing phages (Campbell, 2007). Imprecise excision might be more common with ICEs that have promiscuous integration sites rather than a single preferred site and mainly when they use recombinases with low sequence specificity (Johnson and Grossman, 2015).

Considering all these arguments together, the amplified region observed in clones 1 and 2 is quite atypical and cannot be strictly classified as GI, ICE, AICE, MGE or IME, since this region does not fulfill all the typical characteristics of these elements (Johnson and Grossman, 2015). To explain the amplification of this region we hypothesize that several genes of this region can contribute to the kanamycin resistance by providing secretory pumps that could export the antibiotic. We hypothesized that the high concentration of kanamycin used to select the recombinant clones should force the cells that have integrated a single copy of the kanamycin resistant gene in the chromosome to find additional resistance mechanisms to survive. In this sense, the amplified region contains three MSF transporters proteins (Supplementary Tables S1, S2) that can be used to export kanamycin increasing the antibiotic resistance. Although we do not know precisely the mechanisms that have generated the chromosomal excision of this atypical replicative element and there are still many questions to be answered, the discovery of this phenomenon raises a new scenario regarding the existence of unforeseen mechanisms of evolution in bacteria.

## Data Availability

The raw data supporting the conclusions of this article will be made available by the authors, without undue reservation.

## Footnotes

^1^ http://www.microbesng.uk
