# Supplementary material for: Identification of an atypical replicative genetic element in Rhodococcus jostii RHA1
Source: Front Microbiol. 2025 Jun 2;16:1567901. doi: 10.3389/fmicb.2025.1567901 (PMC12169178; doi:10.3389/fmicb.2025.1567901)
Supplement: Supplementary file 1 [file Supplementary_file_1.docx]

**Additional file 1**

**Identification of an atypical mobile genetic element in *Rhodococcus jostii* RHA1**

Miguel G. Acedos^1,2*^, Isabel de la Torre^1^, Jorge Barriuso^1^, and José L. García^1*^

^1^Department of Plant and Microbial Biotechnology. Centro de Investigaciones Biológicas Margarita Salas. Consejo Superior de Investigaciones Científicas (CSIC). Madrid. Spain

^2^Advanced Biofuels and Bioproducts Unit, Department of Energy. Centro de Investigaciones Energéticas, Medioambientales y Tecnológicas (CIEMAT). Madrid. Spain

*CORRESPONDENCE: M. G. Acedos and J. L. García. [miguel.garciaacedos@ciemat.es](mailto:miguel.garciaacedos@ciemat.es) and jlgarcia@cib.csic.es

**Supplementary Materials and Methods**

**DNA sequencing and assembling**

Genomic DNA libraries were prepared using the Nextera XT Library Prep Kit (Illumina, San Diego, USA), following the manufacturer’s protocol with slight modifications: input DNA was increased twofold, and the PCR elongation time extended to 45 seconds. DNA quantification and library preparation were performed using a Hamilton Microlab STAR automated liquid handling system (Hamilton Bonaduz AG, Switzerland). Libraries were sequenced on an Illumina NovaSeq 6000 platform using a 250 bp paired-end protocol. For samples processed before April 2020, sequencing was done on an Illumina HiSeq 2500 using the same 250 bp paired-end protocol. Raw reads were adapter trimmed using Trimmomatic version 0.30 with a sliding window quality cutoff of Q15. De novo assembly was performed using SPAdes version 3.7, and contigs were annotated with Prokka 1.11. We confirm that no ambiguous bases (Ns) were introduced during assembly, supporting high-quality contigs. Assembly quality was assessed using QUAST. For clone RHA1GI2, the final assembly contained 5480 contigs (2797 ≥ 1 kb), with a total size of 17.4 Mb, N50 of 44,758 bp, and no ambiguous bases (Ns). For clone RHA1GI5, the assembly contained 383 contigs (137 ≥ 1 kb), totaling 9.57 Mb, with an N50 of 156,112 bp. Taxonomic classification using Kraken2 shows that the vast majority of reads (>94%) belong to *Rhodococcus jostii*, with negligible contamination (<1% unclassified, and no reads assigned to Escherichia coli), thus excluding cross-contamination or bacteriophage contamination as a likely source for the observed high coverage contigs.

The relevant statistics for the two analysed clones are summarized.

| Sample ID | Median Insert Size (bp) | Mean Coverage | Number of Reads | Number of Reads with Insert Size > 300 bp | Largest Contig (bp) | N50 (bp) |
| --- | --- | --- | --- | --- | --- | --- |
| 239889_RhodococcusjostiiRHA1_2 | 707 | 78.1 | 2,981,995 | 2,471,468 | 1,162,727 | 44,758 |
| 239890_RhodococcusjostiiRHA1_5 | 714 | 97.4 | 2,010,907 | 1,619,207 | 1,163,941 | 156,112 |

**Supplementary figures and tables**

**Legends to the figures**

**Figure S1.** Map o**f** pNVS vector.

**Figure S2.** Design of *xylABatf1* operon.

**Figure S3**. Map of pNVSxylABatf1 plasmid.

**Figure S4**. Sequences located at the beginning and the end of the mobile element in clones 1 and 2. The sequences of the circularization sites identified by the overlapping reads for both clones are shown in red. Sequences of the region A, B, C and D located at both sides of the circularization sites are shown in grey, blue, yellow and green colours, respectively. Nucleotides underlined in clone 1 indicate a putative reverse complementarity around the circularization site. The nucleotides underlined in clone 2 shows a palindromic sequence in the circularization site.

**Tables**

Table S1. Genes of the mobile element of *R. jostii* RHA1 clone 1

Table S2. Genes of the mobile element of *R. jostii* RHA1 clone 2

Table S3. Annotated recombinases and integrases in *R. jostii* RHA1 genome.


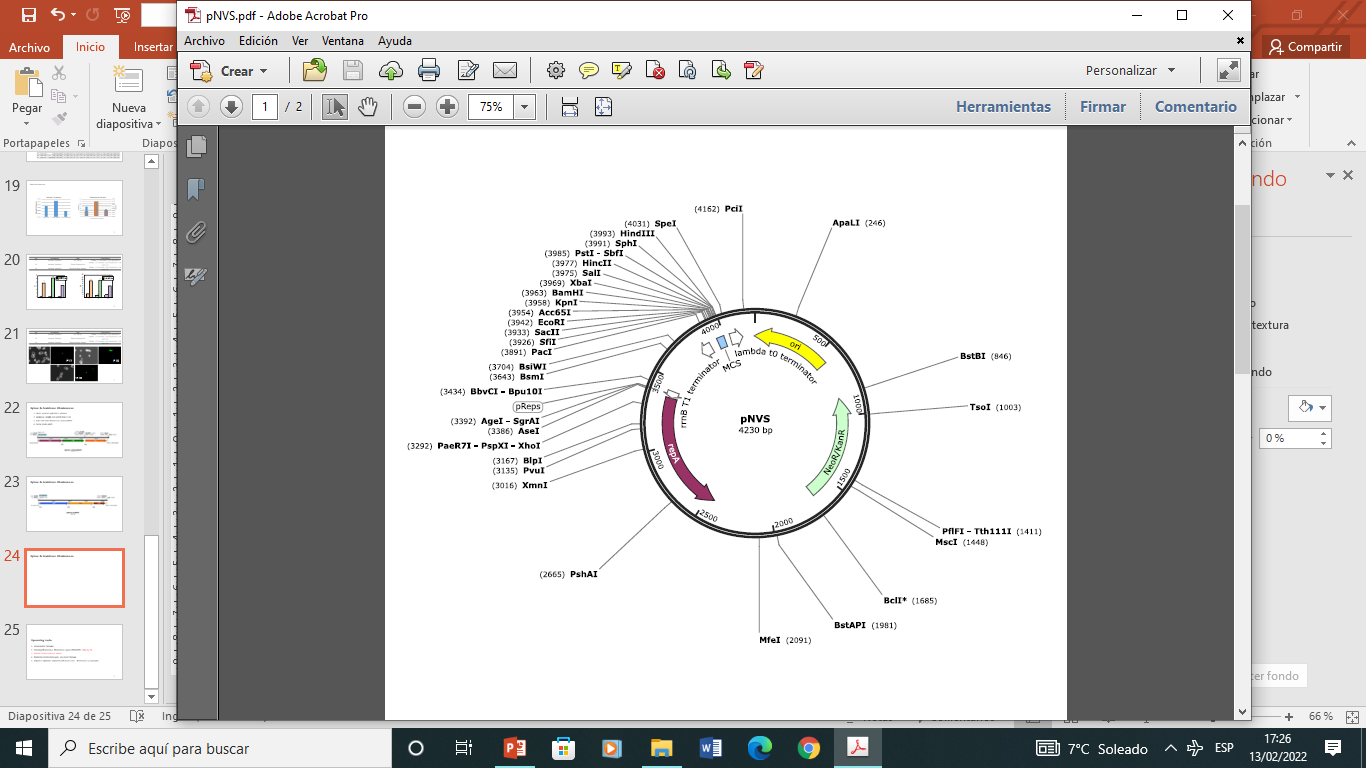


Figure S1.


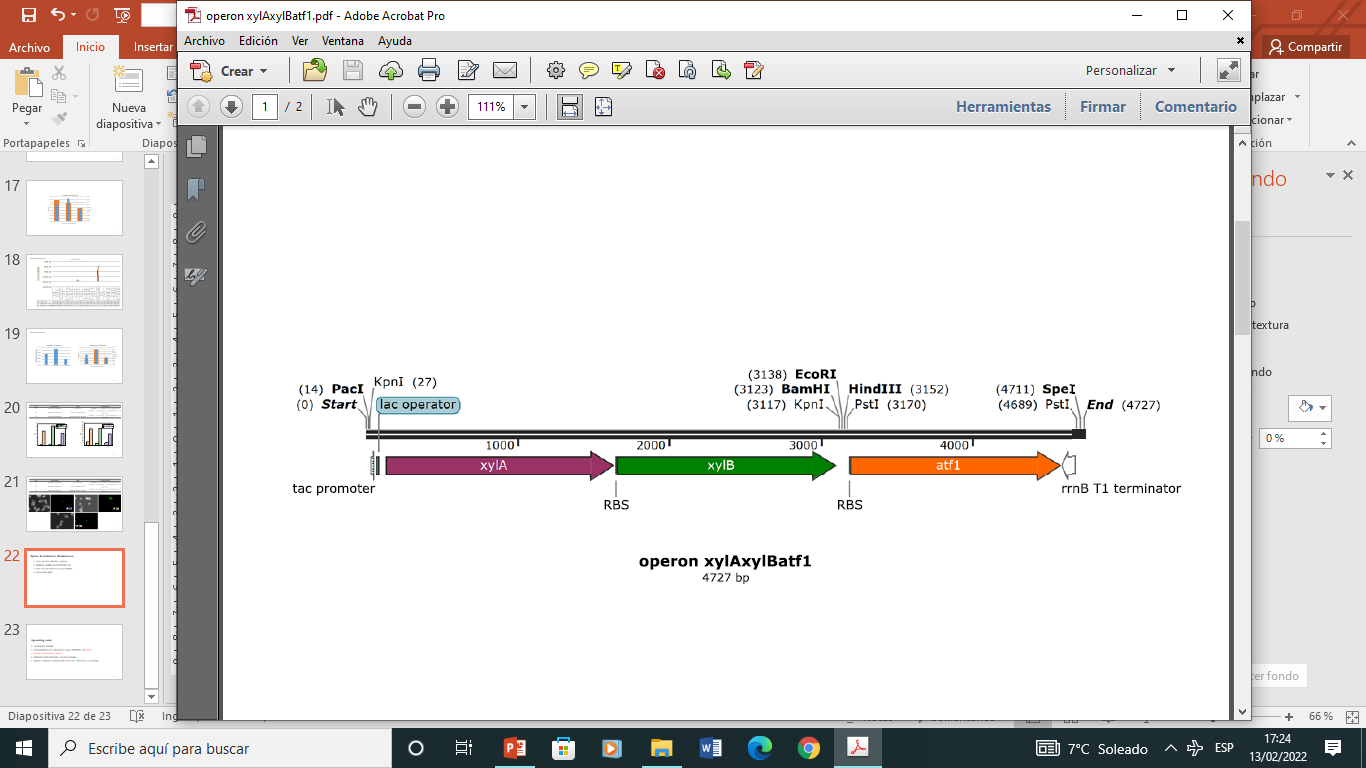


Figure S2


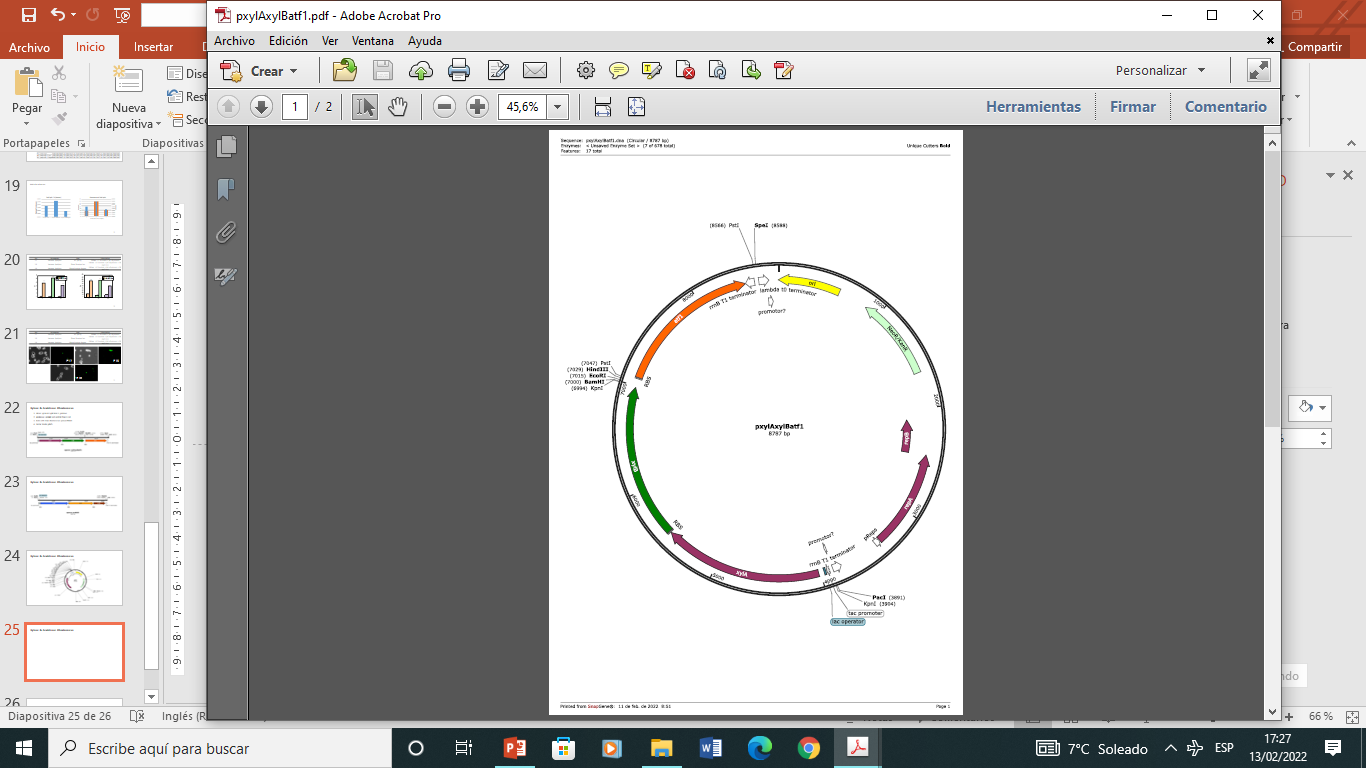


Figure S3.

**Clone 1**

**Region A Region B element Region C Region D**

TCCTGGACGTCGAGCCGCTGCGGATCCTCGACGCGCTGATTCCCGACTTCCAGGCCCAGATCCAGA---------ATGCCGAGCATGTCGCAGGCGCCCAGCACCTGCGGCTCGATGGGCAGGGACTGCTCGTCCACGATC

Region A Region B

TCCTGGACGTCGAGCCGCTGCGGATCCTCGACGCGCTGATTCCCGACTTCCAGGCCCAGATCCAGA

Region C Region D

ATGCCGAGCATGTCGCAGGCGCCCAGCACCTGCGGCTCGATGGGCAGGGACTGCTCGTCCACGATC

Region C Region B

ATGCCGAGCATGTCGCAGGCGCCCAGCACCTGCGGCTGATTCCCGACTTCCAGGCCCAGATCCAGA

(circular)

**Clone 2**

**Region A Region B element Region C Region D**

ACAACCTCGGTATCGAATGGATGCCGCTGCTGCCGGCCATCCTCCTCGCGCTGATCGCGATCGGCGG -------TCCATCATCGCAGGGCCCTCGCTTTCTCGTGACCGGTGTGCGCGGACCAGGCGAGTTCGCCGCCCCG

Region A Region B

ACAACCTCGGTATCGAATGGATGCCGCTGCTGCCGGCCATCCTCCTCGCGCTGATCGCGATCGGCGG

Region C Region D

TCCATCATCGCAGGGCCCTCGCTTTCTCGTGACCGGTGTGCGCGGACCAGGCGAGTTCGCCGCCCCG

Region C Region B

TCCATCATCGCAGGGCCCTCGCTTTCTCGTGACCGGCCATCCTCCTCGCGCTGATCGCGATCGGCGG

(circular)

Figure S4.

Table S1.

| locus_tag | Name | Minimum | Maximum | Length | Direction |
| --- | --- | --- | --- | --- | --- |
| RHA1_RS22560 | ATP-dependent Clp protease proteolytic subunit CDS | 4874757 | 4875332 | 576 | reverse |
| RHA1_RS22555 | hypF CDS | 4872304 | 4874610 | 2307 | reverse |
| RHA1_RS22550 | sedoheptulose 7-phosphate isomerase CDS | 4871510 | 4872259 | 750 | reverse |
| RHA1_RS22545 | SIS domain-containing protein CDS | 4870821 | 4871513 | 693 | reverse |
| RHA1_RS22540 | hypE CDS | 4869724 | 4870824 | 1101 | reverse |
| RHA1_RS22535 | HypC/HybG/HupF family hydrogenase formation chaperone CDS | 4869425 | 4869697 | 273 | reverse |
| RHA1_RS22530 | hypD CDS | 4868272 | 4869399 | 1128 | reverse |
| RHA1_RS22525 | DUF6390 family protein CDS | 4867457 | 4868247 | 791 | reverse |
| RHA1_RS22520 | hypothetical protein CDS | 4866920 | 4867420 | 501 | reverse |
| RHA1_RS22515 | hemerythrin domain-containing protein CDS | 4866320 | 4866892 | 573 | reverse |
| RHA1_RS22510 | ScbA/BarX family gamma-butyrolactone biosynthesis protein CDS | 4865276 | 4866223 | 948 | reverse |
| RHA1_RS22505 | ScbR family autoregulator-binding transcription factor CDS | 4864632 | 4865279 | 648 | forward |
| RHA1_RS22500 | NAD(P)-dependent oxidoreductase CDS | 4863619 | 4864596 | 978 | reverse |
| RHA1_RS22495 | hypothetical protein CDS | 4862691 | 4863482 | 792 | reverse |
| RHA1_RS22490 | HypC/HybG/HupF family hydrogenase formation chaperone CDS | 4862427 | 4862681 | 255 | forward |
| RHA1_RS22485 | hydrogenase maturation protease CDS | 4861911 | 4862423 | 513 | forward |
| RHA1_RS22475 | hypothetical protein CDS | 4860360 | 4861787 | 1428 | forward |
| RHA1_RS22470 | DUF6084 family protein CDS | 4859716 | 4860363 | 648 | forward |
| RHA1_RS22465 | DUF5947 family protein CDS | 4859081 | 4859719 | 639 | forward |
| RHA1_RS22460 | NifU family protein CDS | 4858125 | 4859081 | 957 | forward |
| RHA1_RS22455 | nickel-dependent hydrogenase large subunit CDS | 4856321 | 4858114 | 1794 | forward |
| RHA1_RS22450 | hydrogenase expression protein HypE CDS | 4855208 | 4856263 | 1056 | forward |
| RHA1_RS22445 | hypB CDS | 4854232 | 4855047 | 816 | forward |
| RHA1_RS22440 | hydrogenase maturation nickel metallochaperone HypA CDS | 4853896 | 4854225 | 330 | forward |
| RHA1_RS22435 | GNAT family N-acetyltransferase CDS | 4852493 | 4853755 | 1263 | reverse |
| RHA1_RS22430 | acetyl-CoA C-acetyltransferase CDS | 4851305 | 4852453 | 1149 | forward |
| RHA1_RS22425 | TetR/AcrR family transcriptional regulator CDS | 4850677 | 4851291 | 615 | forward |
| RHA1_RS22420 | SDR family oxidoreductase CDS | 4849782 | 4850585 | 804 | forward |
| RHA1_RS22415 | acyl-CoA dehydrogenase family protein CDS | 4848565 | 4849785 | 1221 | forward |
| RHA1_RS22410 | fadD3 CDS | 4846969 | 4848516 | 1548 | reverse |
| RHA1_RS22405 | enoyl-CoA hydratase CDS | 4846100 | 4846972 | 873 | reverse |
| RHA1_RS22400 | acyl-CoA dehydrogenase family protein CDS | 4844939 | 4846099 | 1161 | reverse |
| RHA1_RS22395 | acyl-CoA dehydrogenase family protein CDS | 4843974 | 4844942 | 969 | reverse |
| RHA1_RS22390 | acyl-CoA/acyl-ACP dehydrogenase CDS | 4842928 | 4843977 | 1050 | reverse |
| RHA1_RS22385 | Cof-type HAD-IIB family hydrolase CDS | 4842061 | 4842876 | 816 | forward |
| RHA1_RS22380 | aldo/keto reductase CDS | 4841115 | 4841996 | 882 | reverse |
| RHA1_RS22375 | cytochrome P450 CDS | 4839871 | 4841046 | 1176 | forward |
| RHA1_RS22370 | SDR family oxidoreductase CDS | 4838878 | 4839729 | 852 | reverse |
| RHA1_RS22365 | acetoacetate decarboxylase family protein CDS | 4838153 | 4838881 | 729 | reverse |
| RHA1_RS22360 | neutral/alkaline ceramidase CDS | 4836000 | 4838030 | 2031 | reverse |
| RHA1_RS22355 | amino acid-binding protein CDS | 4835004 | 4835852 | 849 | reverse |
| RHA1_RS22350 | YncE family protein CDS | 4833980 | 4834951 | 972 | reverse |
| RHA1_RS22345 | MFS transporter CDS | 4832778 | 4833977 | 1200 | forward |
| RHA1_RS22340 | MarR family transcriptional regulator CDS | 4832179 | 4832622 | 444 | reverse |
| RHA1_RS22335 | thpD CDS | 4831223 | 4832137 | 915 | forward |
| RHA1_RS22330 | GntR family transcriptional regulator CDS | 4830509 | 4831186 | 678 | forward |
| RHA1_RS22325 | maleate cis-trans isomerase CDS | 4829782 | 4830522 | 741 | forward |
| RHA1_RS22320 | hypothetical protein CDS | 4829004 | 4829747 | 744 | forward |
| RHA1_RS22315 | D-2-hydroxyacid dehydrogenase CDS | 4827949 | 4828923 | 975 | reverse |
| RHA1_RS22310 | amidase CDS | 4826542 | 4827912 | 1371 | reverse |
| RHA1_RS22305 | protein kinase family protein CDS | 4824911 | 4826401 | 1491 | reverse |
| RHA1_RS22300 | DUF202 domain-containing protein CDS | 4824567 | 4824914 | 348 | reverse |
| RHA1_RS22295 | SUMF1/EgtB/PvdO family nonheme iron enzyme CDS | 4823691 | 4824575 | 885 | forward |
| RHA1_RS22290 | haloacid dehalogenase-like hydrolase CDS | 4822672 | 4823616 | 945 | forward |
| RHA1_RS22285 | arylsulfatase CDS | 4821128 | 4822666 | 1539 | forward |
| RHA1_RS22280 | hypothetical protein CDS | 4820367 | 4821020 | 654 | forward |
| RHA1_RS22275 | treS CDS | 4818103 | 4820306 | 2204 | forward |
| RHA1_RS22265 | dihydrodipicolinate synthase family protein CDS | 4817391 | 4817684 | 294 | reverse |
| RHA1_RS22260 | proline racemase family protein CDS | 4816175 | 4817209 | 1035 | reverse |
| RHA1_RS22255 | LLM class flavin-dependent oxidoreductase CDS | 4815247 | 4816131 | 885 | forward |
| RHA1_RS22250 | substrate-binding domain-containing protein CDS | 4814127 | 4815200 | 1074 | forward |
| RHA1_RS22245 | ThuA domain-containing protein CDS | 4813243 | 4813908 | 666 | reverse |
| RHA1_RS22240 | thiamine pyrophosphate-binding protein CDS | 4811435 | 4813222 | 1788 | reverse |
| RHA1_RS22235 | GMC family oxidoreductase CDS | 4809852 | 4811438 | 1587 | reverse |
| RHA1_RS22230 | MFS transporter CDS | 4808559 | 4809779 | 1221 | forward |
| RHA1_RS22225 | MFS transporter CDS | 4807156 | 4808445 | 1290 | forward |
| RHA1_RS22220 | PucR family transcriptional regulator CDS | 4805401 | 4806942 | 1542 | forward |
| RHA1_RS22215 | hisD CDS | 4803943 | 4805268 | 1326 | reverse |
| RHA1_RS22210 | aspartate ammonia-lyase CDS | 4802552 | 4803946 | 1395 | reverse |
| RHA1_RS22205 | GMC family oxidoreductase CDS | 4800793 | 4802379 | 1587 | reverse |
| RHA1_RS22200 | thiamine pyrophosphate-binding protein CDS | 4798958 | 4800757 | 1800 | reverse |
| RHA1_RS22195 | sugar phosphate isomerase/epimerase CDS | 4798106 | 4798945 | 840 | reverse |
| RHA1_RS22180 | substrate-binding domain-containing protein CDS | 4795408 | 4796463 | 1056 | reverse |
| RHA1_RS22175 | LysR family transcriptional regulator CDS | 4794513 | 4795406 | 894 | forward |

Table S2.

|  | Name | Minimum | Maximum | Length | Direction |
| --- | --- | --- | --- | --- | --- |
| RHA1_RS22640 | hutG CDS | 4889141 | 4890103 | 963 | reverse |
| RHA1_RS22635 | alpha/beta hydrolase CDS | 4888286 | 4889119 | 834 | forward |
| RHA1_RS22630 | cold-shock protein CDS | 4887850 | 4888053 | 204 | reverse |
| RHA1_RS22625 | hypothetical protein CDS | 4887377 | 4887751 | 375 | forward |
| RHA1_RS22620 | acyl-CoA dehydrogenase family protein CDS | 4886042 | 4887193 | 1152 | reverse |
| RHA1_RS22615 | CoA transferase CDS | 4884882 | 4886030 | 1149 | reverse |
| RHA1_RS22610 | helix-turn-helix domain-containing protein CDS | 4884182 | 4884844 | 663 | reverse |
| RHA1_RS22605 | LysR family transcriptional regulator CDS | 4883284 | 4884174 | 891 | forward |
| RHA1_RS22600 | sodium:solute symporter CDS | 4881603 | 4883078 | 1476 | reverse |
| RHA1_RS22595 | TetR/AcrR family transcriptional regulator CDS | 4880925 | 4881560 | 636 | reverse |
| RHA1_RS22590 | C-terminal binding protein CDS | 4879969 | 4880928 | 960 | reverse |
| RHA1_RS22585 | class II aldolase/adducin family protein CDS | 4879162 | 4879944 | 783 | forward |
| RHA1_RS22580 | TetR/AcrR family transcriptional regulator CDS | 4878502 | 4879086 | 585 | forward |
| RHA1_RS22575 | cytochrome P450 CDS | 4877007 | 4878404 | 1398 | reverse |
| RHA1_RS22570 | lhgO CDS | 4875730 | 4876935 | 1206 | forward |
| RHA1_RS22565 | helix-turn-helix domain-containing protein CDS | 4875413 | 4875685 | 273 | reverse |
| RHA1_RS22560 | ATP-dependent Clp protease proteolytic subunit CDS | 4874757 | 4875332 | 576 | reverse |
| RHA1_RS22555 | hypF CDS | 4872304 | 4874610 | 2307 | reverse |
| RHA1_RS22550 | sedoheptulose 7-phosphate isomerase CDS | 4871510 | 4872259 | 750 | reverse |
| RHA1_RS22545 | SIS domain-containing protein CDS | 4870821 | 4871513 | 693 | reverse |
| RHA1_RS22540 | hypE CDS | 4869724 | 4870824 | 1101 | reverse |
| RHA1_RS22535 | HypC/HybG/HupF family hydrogenase formation chaperone CDS | 4869425 | 4869697 | 273 | reverse |
| RHA1_RS22530 | hypD CDS | 4868272 | 4869399 | 1128 | reverse |
| RHA1_RS22525 | DUF6390 family protein CDS | 4867457 | 4868247 | 791 | reverse |
| RHA1_RS22520 | hypothetical protein CDS | 4866920 | 4867420 | 501 | reverse |
| RHA1_RS22515 | hemerythrin domain-containing protein CDS | 4866320 | 4866892 | 573 | reverse |
| RHA1_RS22510 | ScbA/BarX family gamma-butyrolactone biosynthesis protein CDS | 4865276 | 4866223 | 948 | reverse |
| RHA1_RS22505 | ScbR family autoregulator-binding transcription factor CDS | 4864632 | 4865279 | 648 | forward |
| RHA1_RS22500 | NAD(P)-dependent oxidoreductase CDS | 4863619 | 4864596 | 978 | reverse |
| RHA1_RS22495 | hypothetical protein CDS | 4862691 | 4863482 | 792 | reverse |
| RHA1_RS22490 | HypC/HybG/HupF family hydrogenase formation chaperone CDS | 4862427 | 4862681 | 255 | forward |
| RHA1_RS22485 | hydrogenase maturation protease CDS | 4861911 | 4862423 | 513 | forward |
| RHA1_RS22475 | hypothetical protein CDS | 4860360 | 4861787 | 1428 | forward |
| RHA1_RS22470 | DUF6084 family protein CDS | 4859716 | 4860363 | 648 | forward |
| RHA1_RS22465 | DUF5947 family protein CDS | 4859081 | 4859719 | 639 | forward |
| RHA1_RS22460 | NifU family protein CDS | 4858125 | 4859081 | 957 | forward |
| RHA1_RS22455 | nickel-dependent hydrogenase large subunit CDS | 4856321 | 4858114 | 1794 | forward |
| RHA1_RS22450 | hydrogenase expression protein HypE CDS | 4855208 | 4856263 | 1056 | forward |
| RHA1_RS22445 | hypB CDS | 4854232 | 4855047 | 816 | forward |
| RHA1_RS22440 | hydrogenase maturation nickel metallochaperone HypA CDS | 4853896 | 4854225 | 330 | forward |
| RHA1_RS22435 | GNAT family N-acetyltransferase CDS | 4852493 | 4853755 | 1263 | reverse |
| RHA1_RS22430 | acetyl-CoA C-acetyltransferase CDS | 4851305 | 4852453 | 1149 | forward |
| RHA1_RS22425 | TetR/AcrR family transcriptional regulator CDS | 4850677 | 4851291 | 615 | forward |
| RHA1_RS22420 | SDR family oxidoreductase CDS | 4849782 | 4850585 | 804 | forward |
| RHA1_RS22415 | acyl-CoA dehydrogenase family protein CDS | 4848565 | 4849785 | 1221 | forward |
| RHA1_RS22410 | fadD3 CDS | 4846969 | 4848516 | 1548 | reverse |
| RHA1_RS22405 | enoyl-CoA hydratase CDS | 4846100 | 4846972 | 873 | reverse |
| RHA1_RS22400 | acyl-CoA dehydrogenase family protein CDS | 4844939 | 4846099 | 1161 | reverse |
| RHA1_RS22395 | acyl-CoA dehydrogenase family protein CDS | 4843974 | 4844942 | 969 | reverse |
| RHA1_RS22390 | acyl-CoA/acyl-ACP dehydrogenase CDS | 4842928 | 4843977 | 1050 | reverse |
| RHA1_RS22385 | Cof-type HAD-IIB family hydrolase CDS | 4842061 | 4842876 | 816 | forward |
| RHA1_RS22380 | aldo/keto reductase CDS | 4841115 | 4841996 | 882 | reverse |
| RHA1_RS22375 | cytochrome P450 CDS | 4839871 | 4841046 | 1176 | forward |
| RHA1_RS22370 | SDR family oxidoreductase CDS | 4838878 | 4839729 | 852 | reverse |
| RHA1_RS22365 | acetoacetate decarboxylase family protein CDS | 4838153 | 4838881 | 729 | reverse |
| RHA1_RS22360 | neutral/alkaline ceramidase CDS | 4836000 | 4838030 | 2031 | reverse |
| RHA1_RS22355 | amino acid-binding protein CDS | 4835004 | 4835852 | 849 | reverse |
| RHA1_RS22350 | YncE family protein CDS | 4833980 | 4834951 | 972 | reverse |
| RHA1_RS22345 | MFS transporter CDS | 4832778 | 4833977 | 1200 | forward |
| RHA1_RS22340 | MarR family transcriptional regulator CDS | 4832179 | 4832622 | 444 | reverse |
| RHA1_RS22335 | thpD CDS | 4831223 | 4832137 | 915 | forward |
| RHA1_RS22330 | GntR family transcriptional regulator CDS | 4830509 | 4831186 | 678 | forward |
| RHA1_RS22325 | maleate cis-trans isomerase CDS | 4829782 | 4830522 | 741 | forward |
| RHA1_RS22320 | hypothetical protein CDS | 4829004 | 4829747 | 744 | forward |
| RHA1_RS22315 | D-2-hydroxyacid dehydrogenase CDS | 4827949 | 4828923 | 975 | reverse |
| RHA1_RS22310 | amidase CDS | 4826542 | 4827912 | 1371 | reverse |
| RHA1_RS22305 | protein kinase family protein CDS | 4824911 | 4826401 | 1491 | reverse |
| RHA1_RS22300 | DUF202 domain-containing protein CDS | 4824567 | 4824914 | 348 | reverse |
| RHA1_RS22295 | SUMF1/EgtB/PvdO family nonheme iron enzyme CDS | 4823691 | 4824575 | 885 | forward |
| RHA1_RS22290 | haloacid dehalogenase-like hydrolase CDS | 4822672 | 4823616 | 945 | forward |
| RHA1_RS22285 | arylsulfatase CDS | 4821128 | 4822666 | 1539 | forward |
| RHA1_RS22280 | hypothetical protein CDS | 4820367 | 4821020 | 654 | forward |
| RHA1_RS22275 | treS CDS | 4818103 | 4820306 | 2204 | forward |
| RHA1_RS22265 | dihydrodipicolinate synthase family protein CDS | 4817391 | 4817684 | 294 | reverse |
| RHA1_RS22260 | proline racemase family protein CDS | 4816175 | 4817209 | 1035 | reverse |
| RHA1_RS22255 | LLM class flavin-dependent oxidoreductase CDS | 4815247 | 4816131 | 885 | forward |
| RHA1_RS22250 | substrate-binding domain-containing protein CDS | 4814127 | 4815200 | 1074 | forward |
| RHA1_RS22245 | ThuA domain-containing protein CDS | 4813243 | 4813908 | 666 | reverse |
| RHA1_RS22240 | thiamine pyrophosphate-binding protein CDS | 4811435 | 4813222 | 1788 | reverse |
| RHA1_RS22235 | GMC family oxidoreductase CDS | 4809852 | 4811438 | 1587 | reverse |
| RHA1_RS22230 | MFS transporter CDS | 4808559 | 4809779 | 1221 | forward |
| RHA1_RS22225 | MFS transporter CDS | 4807156 | 4808445 | 1290 | forward |

Table S3.

| **GENE** | **PUTATIVE FUNCTION** |
| --- | --- |
| *RHA1_ro00178* | serine recombinase |
| *RHA1_ro00464* | tyrosine recombinase |
| *RHA1_ro00671* | recombinase |
| *RHA1_ro00673* | tyrosine recombinase |
| *RHA1_ro00929* | tyrosine recombinase |
| *RHA1_ro00929* | tyrosine recombinase |
| *RHA1_ro03118* | tyrosine recombinase |
| *RHA1_ro03177* | tyrosine recombinase |
| *RHA1_ro06554* | integrase |
| *RHA1_ro06554* | integrase |
| *RHA1_ro06765* | recombinase A |
| *RHA1_ro06911* | integrase/recombinase |
| *RHA1_ro06911* | integrase/recombinase |
| *RHA1_ro08013* | integrase |
| *RHA1_ro08109* | integrase/recombinase |
| *RHA1_ro08109* | integrase/recombinase |
| *RHA1_ro08247* | serine recombinase |
| *RHA1_ro08256* | serine recombinase |
| *RHA1_ro08301* | integrase/recombinase |
| *RHA1_ro08301* | integrase/recombinase |
| *RHA1_ro08484* | integrase/recombinase |
| *RHA1_ro08484* | integrase/recombinase |
| *RHA1_ro08485* | integrase/recombinase |
| *RHA1_ro08485* | integrase/recombinase |
| *RHA1_ro08486* | integrase/recombinase |
| *RHA1_ro08486* | integrase/recombinase |
| *RHA1_ro08525* | site-specific DNA recombinase/resolvase |
| *RHA1_ro08541* | transposase/integrase |
| *RHA1_ro08606* | integrase |
| *RHA1_ro08779* | integrase/recombinase |
| *RHA1_ro08779* | integrase/recombinase |
| *RHA1_ro09129* | site-specific recombinase, phage integrase family protein |
| *RHA1_ro09129* | site-specific recombinase, phage integrase family protein |
| *RHA1_ro10101* | integrase/recombinase |
| *RHA1_ro10101* | integrase/recombinase |
| *RHA1_ro10102* | integrase/recombinase |
| *RHA1_ro10102* | integrase/recombinase |
| *RHA1_ro10275* | integrase/recombinase |
| *RHA1_ro10275* | integrase/recombinase |
| *RHA1_ro10276* | DNA integrase/recombinase |
| *RHA1_ro10276* | DNA integrase/recombinase |
| *RHA1_ro10439* | integrase/recombinase, XerD and XerC family |
| *RHA1_ro10439* | integrase/recombinase, XerD and XerC family |
| *RHA1_ro11038* | integrase |
| *RHA1_ro11144* | integrase/recombinase, XerC and XerD family |
